# Ileal Dieulafoy lesion arose 15 years after partial small bowel resection for meconium obstruction of the neonate: a case report

**DOI:** 10.1186/s12887-021-02914-7

**Published:** 2021-10-07

**Authors:** Maho Iwamoto, Tsugumichi Koshinaga, Eri Fujita, Manabu Hanada, Shuichiro Uehara, Mitsuhiko Moriyama

**Affiliations:** 1 Division of Gastroenterology and Hepatology, Department of Internal Medicine, Nihon University School of Medicine, 30-1 Oyaguchi-Kamicho, Itabashi-ku, Tokyo, 173-8610 Japan; 2 Department of Pediatric Surgery, Nihon University School of Medicine, 30-1 Oyaguchi-Kamicho, Itabashi-ku, Tokyo, 173-8610 Japan

**Keywords:** Ileal Dieulafoy lesion, Perianastomotic ulcer, Meconium obstruction, Small bowel resection, Case report

## Abstract

**Background:**

Anastomotic or perianastomotic ulcers present with symptoms such as chronic anaemia and occult bleeding as long-term complications of bowel resection performed in infancy.

**Case presentation:**

Herein, we describe a 15-year-old girl with a history of surgery for meconium obstruction without mucoviscidosis in infancy who was hospitalized with chief complaints of presyncope and convulsions. Seven hours after admission, she developed melena and went into shock. An emergency laparotomy was performed, and a Dieulafoy lesion was detected near the site of ileal anastomosis from the surgery that had been performed during infancy.

**Conclusions:**

Although overt massive lower gastrointestinal bleeding necessitating emergency care is rare in the long term after infant bowel resection, Dieulafoy lesions can cause serious bleeding, requiring rapid life-saving haemostatic procedures.

**Supplementary Information:**

The online version contains supplementary material available at 10.1186/s12887-021-02914-7.

## Background

Among the long-term complications of infant bowel resection, including necrotizing enterocolitis (NEC), anastomotic and perianastomotic lesions are relatively rare [1]. Anastomotic and perianastomotic ulcers are usually detected during investigations for chronic anaemia or faecal occult blood, and the diagnosis is often delayed by several years. However, in rare cases, ulceration may develop rapidly with massive life-threatening bleeding requiring emergency treatment [2].

Dieulafoy lesions are detected as protruding vessels within a minute mucosal defect or within normal surrounding mucosa. Furthermore, Dieulafoy lesions in the small bowel are uncommon in both children and adults. However, all cases present with overt bleeding, necessitating blood transfusions for serious lower gastrointestinal bleeding that generally requires rapid life-saving haemostatic procedures [3]. We report a 15-year-old girl with a history of surgery for meconium obstruction without mucoviscidosis in infancy who rapidly developed massive bleeding from an ileal perianastomotic Dieulafoy lesion and required emergency laparotomy for haemostatic procedures.

## Case presentation

A 15-year-old girl born prematurely with a low birth weight (696 g) developed meconium obstruction without mucoviscidosis when she was 5 days old. A 3-cm section of her small intestine, including a stenotic lesion 35 cm from the ligament of Treitz, was resected. An ileostomy was performed 65 cm from the ligament of Treitz. The ileostomy was reversed 6 months later, with no further complications.

**Fig. 1:**
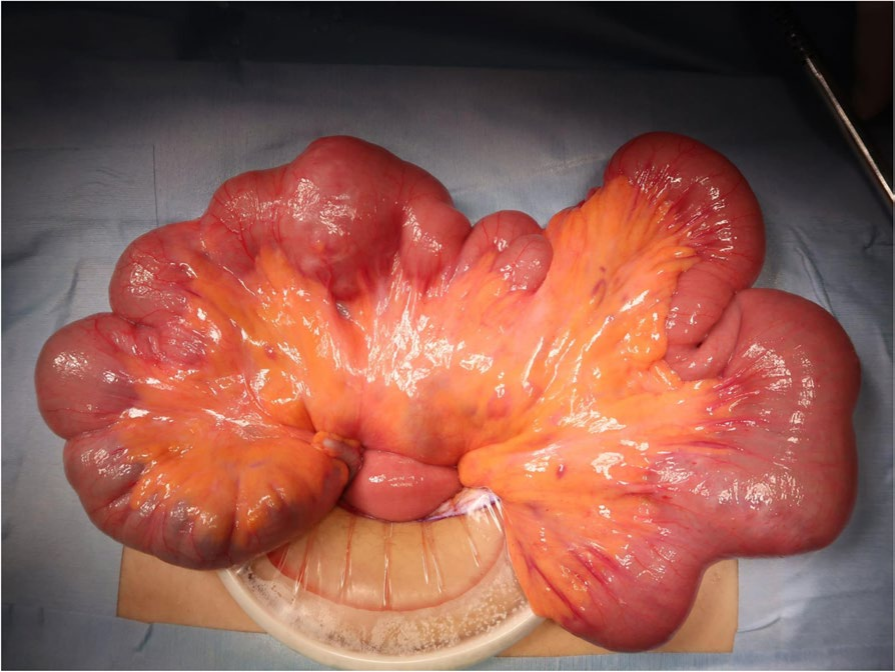
Surgical findings: Irregular intestinal dilation and dark-red stagnant intestinal content were observed in the ileum 40 cm from the ileocecal junction. A scar assumed to be the anastomotic site from prior surgery was noted at the same site

**Fig. 2:**
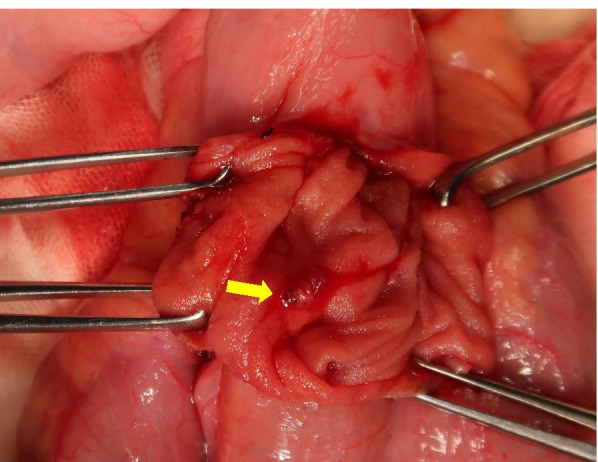
A 2-cm incision made along the long axis revealed microulceration with exposed vessels on the mesentery and persistent bleeding. Since suturing alone was not sufficient to achieve haemostasis, a cuneiform incision was made at the same site, allowing haemostasis to be achieved

**Fig. 3:**
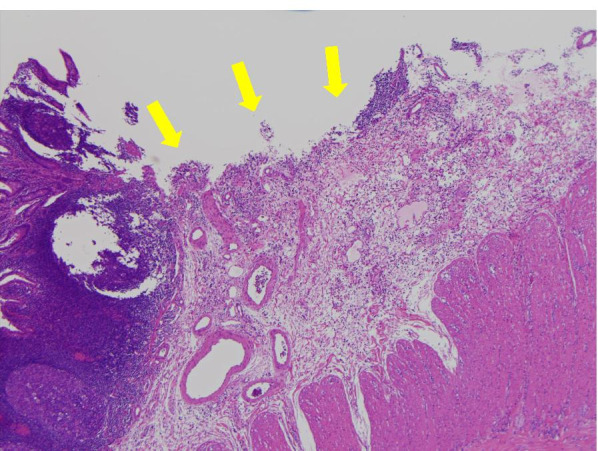
Intraoperative histopathological findings: A 25 × 20 × 10 mm specimen of the small bowel consistent with ulcer; HE 4X magnification. Microulceration, with a partial epithelial defect and inflammatory cell infiltration, was detected. A slightly dilated artery was identified in the submucosal layer [yellow arrow] HE: haematoxylin and eosin staining.

A day prior to the current presentation, the patient experienced intermittent lower abdominal pain and was emergently transported to a local hospital with presyncope and convulsions, which both occurred at 07:00 on the day of the consultation. There were no abnormal findings on head computed tomography (CT) or abdominal ultrasonography. Her haemoglobin level was 11.8 g/dL (reference range: 11.6–14.8 g/dL). The patient was sent home. Massive melena occurred at approximately 14:00. Furthermore, she had difficulty maintaining an upright position and was therefore again urgently transported to the local hospital. Her haemoglobin level had decreased to 6.7 g/dL over the 7-h period since her morning visit to the hospital. Although blood transfusion was started, massive melena persisted, and she was transferred to our hospital at 23:00. The patient’s height and weight were 160 cm and 45 kg, respectively. Her Glasgow Coma Scale score was 14/15 (eye opening 3, best verbal response 5, best motor response 6), her blood pressure was 109/51 mmHg, and her pulse was 76 bpm in the Trendelenburg position. However, her blood pressure dropped to 73/36 mmHg, her consciousness further decreased, and she went into shock during the examination. Mild oppressive pain in the lower abdomen was noted. There was no increase in C-reactive protein, and the patient also tested negative for coronavirus, norovirus, adenovirus, and faecal bacteria. Contrast-enhanced abdominopelvic CT examination showed massive bleeding in the colon, but the source was not identifiable. The patient was subsequently diagnosed with haemorrhagic shock due to lower gastrointestinal bleeding. Thereafter, emergency endoscopy was performed under general anaesthesia. In the operating room, systolic blood pressure dropped into the 60s, necessitating an intraoperative transfusion of 1600 mL of blood. Although no abnormalities were detected in the upper gastrointestinal tract, lower gastrointestinal endoscopy revealed massive blood flow from the terminal ileum, and the patient was diagnosed with small bowel haemorrhage. The decision was made to perform open surgery. Intraoperative findings included irregular dilation in the ileum 40 cm from the ileocecal junction, and a scar presumed to be the anastomotic site was also noted (Fig. 1). Dark red stagnant intestinal content was clearly observed on fluoroscopy. An incision made in the direction of the long axis revealed microulceration with exposed vessels on the mesentery and persistent bleeding (Fig. 2). Since suturing alone was not sufficient to achieve haemostasis, a cuneiform incision was made at the same site, and haemostasis was achieved. Histopathological examination of the surgical specimen showed microulceration with a partial epithelial defect and inflammatory cell infiltration (Fig. 3). No abnormalities were observed in the surrounding membranes. An ileal Dieulafoy lesion was diagnosed based on the appearance of slightly distended arteries and veins in the submucosa of the ulcerated site. The postoperative course was uneventful. The patient was discharged on day 11 of hospitalization. She recovered well enough to participate in her school’s athletic competition 2 months later.

## Discussion and conclusions

Acute massive lower gastrointestinal bleeding, as in our present patient, is a rare long-term complication after infant bowel resection.

We treated a 15-year-old girl who had experienced rapid onset of massive bleeding from an ileal perianastomotic Dieulafoy lesion and required an emergency laparotomy for haemostatic procedures. She had a history of surgery for meconium obstruction without mucoviscidosis in infancy. It is important to keep anastomotic or perianastomotic ulcers in mind in patients who have a history of intestinal resection during the neonatal period.

The incidence of symptomatic anastomotic ulcers in the paediatric population is approximately 0.3%, and such ulcers are a relatively rare complication of bowel resection [1]. However, anastomotic or perianastomotic ulcers are known to cause chronic anaemia and occult bleeding, which are both potential long-term complications after bowel resection in infancy. Sudden massive lower gastrointestinal bleeding is rare. However, Dieulafoy lesions can produce serious lower gastrointestinal bleeding requiring rapid life-saving haemostatic procedures [2].

As our patient initially presented with presyncope and convulsions, gastrointestinal bleeding was not suspected at the first visit. However, melena manifested 7 h later, and she went into shock 9 h thereafter. This patient’s course showed that a perianastomotic Dieulafoy lesion can be life-threatening and that an urgent haemostatic procedure is required. Sudden massive bleeding might render children especially susceptible to haemodynamic instability, and the onset may have an atypical course. Caution is thus required in patients with a history of abdominal surgery in the neonatal period, even those who have been well over the long term since the operation.

To our knowledge, this is the second report describing a patient who developed sudden massive lower gastrointestinal bleeding long after surgery performed in infancy [2].

Our search of PubMed from 1992 to 2020 for anastomotic or perianastomotic ulcers in paediatric populations, with the search terms “anastomotic ulcers”, “perianastomotic ulcers”, “infant” and “neonatal”, yielded nine reports in English describing the symptoms of these lesions (Table 1) [1, 2, 4–10]. Of the 35 cases presented in Table 1, including ours, two had a sudden onset, while the onset was more gradual in the other cases. Of all the cases, 60% had no symptoms of overt bleeding, 66% eventually had to undergo surgery, and 59% developed recurrences during follow-up. Some reports described little or no follow-up after the operation performed in infancy, but this may not be clinically appropriate. Given the high percentage of recurrent cases, regular follow-up is highly recommended for children who undergo surgery in infancy [2].

**Table 1:** Reports of anastomotic or perianastomotic ulcers after infant bowel resection in children

Case	Author	Primary condition	Symptoms at onset	Final treatment	Recurrence while being treated
1	Hamilton	NEC	Anaemia, FOB	Surgery	Yes
2		Gastroschisis	Abdominal pain, melena, IDA	Surgery	No
3	Paterson	NEC	IDA	Medication	No
4	Bhargava	NEC	Anaemia, FOB	Surgery	No
5	Sondheimer	NEC	Acute lower GI bleeding	Surgery	Yes
6		NEC	Acute lower GI bleeding	Medication	N/A
7		NEC	Abdominal pain, diarrhoea, FOB	Surgery	Yes
8		NEC	Abdominal pain, diarrhoea, FOB	Surgery	Yes
9		NEC, hernia	Anaemia, FOB	Surgery	No
10		NEC	Anaemia, FOB	Surgery	Yes
11	Arnbjornsson	Gastroschisis, atresia	Loose stool, anaemia, melena	Medication	Yes
12	Ceylan	NEC	Anaemia, FOB	Surgery	Yes
13		Gastroschisis	Abdominal pain, melena, IDA	Surgery	Yes
14	Charbit-Henrion	Gastroschisis	IDA	Medication	Yes
15		NEC	IDA	Surgery	Yes
16		Gastroschisis, NEC	Asthenia, IDA	Surgery	Yes
17		Gastroschisis	IDA, rectal bleeding	Surgery	Yes
18		Gastroschisis	IDA	Surgery	Yes
19		Bladder extropy	IDA	None	No
20		Atresia	Acute IDA	Surgery	Yes
21		Atresia	Acute IDA	Surgery	Yes
22		NEC	IDA	Surgery	Yes
23	Bass	NEC	IDA, FOB	Medication	No
24		Atresia	Lower GI bleeding	Surgery	Yes
25		NEC	Lower GI bleeding, anaemia	Surgery	No
26		Gastroschisis	GI bleeding, anaemia	Medication	Yes
27	Fusaro	Gastroschisis	IDA, FOB	Medication	No
28		NEC	IDA, rectal bleeding	Endoscopy	No
29		NEC	IDA, FOB	Endoscopy	No
30		NEC	IDA, melena	Surgery	Yes
31		NEC	Rectal bleeding	Endoscopy	No
32		Gastroschisis	IDA, FOB	Surgery	No
33		Atresia	IDA, rectal bleeding	Medication	No
34		NEC	IDA, FOB	Surgery	Yes
35	Our case	Meconium disease	Presyncope, convulsions, melena	Surgery	No

**Abbreviations:** FOB test: faecal occult blood test, IDA: iron-deficiency anaemia, NEC: necrotizing enterocolitis, N/A: not available

Video capsule endoscopy and double-balloon endoscopy have both been reported to be safe and effective in children as endoscopy methods for the small intestine. Video capsule endoscopy might be useful as postoperative follow-up for children because it is easy and noninvasive, and a patency capsule test can reduce the risk of retention [10–12].

The pathogenesis of Dieulafoy lesions involves both congenital and acquired factors, such as abnormal distribution and dilation of submucosal capillaries, microaneurysms, and arteriovenous malformations combined with mucosal injury secondary to mechanical factors and other causes [13, 14].

The pathogenesis of the lesion in our present case was a perianastomotic site from the surgery performed in infancy. The patient also suffered from intermittent lower abdominal pain the day before the visit, suggesting microulceration caused by mechanical triggers as well as changes in the vascular distribution near the anastomotic site as the probable underlying causes. Moreover, we suspected that intestinal ischaemia occurred during the operation performed when she was an infant.

We conclude that although sudden massive lower gastrointestinal bleeding requiring emergency care is a rare long-term complication after infant bowel resection, Dieulafoy lesions can produce serious bleeding requiring rapid life-saving haemostatic procedures. Long-term follow-up is recommended after surgery in infancy due to the high percentage of recurrences of anastomotic ulcers.

## Supplementary Information

**ESM 1.**

## Data Availability

The data and materials used and/or analysed during the current study are presented within the manuscript. Any further requests regarding the study data should be addressed to the corresponding author.

## Abbreviations

CT: computed tomography
NEC: necrotizing enterocolitis
