# Supplementary material for: Ileal Dieulafoy lesion arose 15 years after partial small bowel resection for meconium obstruction of the neonate: a case report
Source: BMC Pediatr. 2021 Oct 7;21:437. doi: 10.1186/s12887-021-02914-7 (PMC8494758; doi:10.1186/s12887-021-02914-7)
Supplement: Supplementary file 1 — ESM 1. [file 12887_2021_2914_MOESM1_ESM.docx]

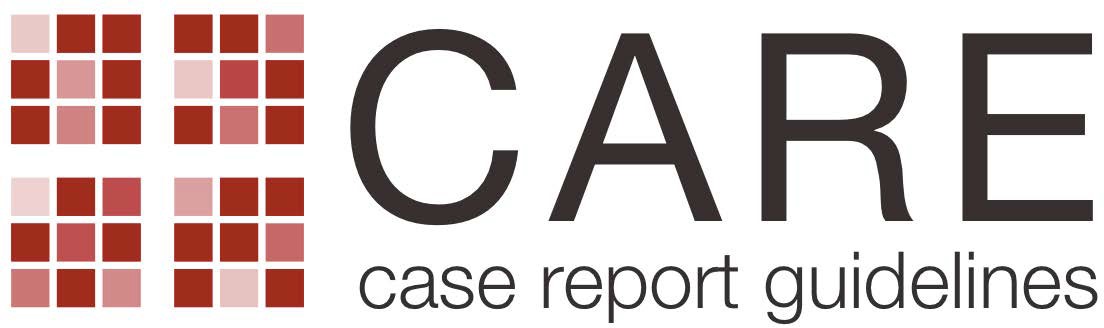
CARE Checklist of information to include when writing a case report
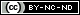


**Topic Item Checklist item description Reported on Line**

**Title 1** The diagnosis or intervention of primary focus followed by the words “case report” Page 1, title

**Key Words 2** 2 to 5 key words that identify diagnoses or interventions in this case report, including "case report" Page 2, key words

**Abstract**

**(no references)**

**3a** Introduction: What is unique about this case and what does it add to the scientific literature? Background, paragraph 1

**3b** Main symptoms and/or important clinical findings Case presentation, paragraph 1

**3c** The main diagnoses, therapeutic interventions, and outcomes Case presentation, paragraph 1

**3d** Conclusion—What is the main “take-away” lesson(s) from this case? Conclusion, paragraph 1

**Introduction 4** One or two paragraphs summarizing why this case is unique (**may include references**) Background, paragraph 1,2

**Patient Information 5a** De-identified patient specific information Case presentation, paragraph 1,2

**5b** Primary concerns and symptoms of the patient Case presentation, paragraph 2

**5c** Medical, family, and psycho-social history including relevant genetic information Case presentation, paragraph 1

**5d** Relevant past interventions with outcomes Case presentation, paragraph 1

**Clinical Findings**

**Timeline**

**Diagnostic Assessment**

**Therapeutic Intervention**

**Follow-up and Outcomes**

1. Describe significant physical examination (PE) and important clinical findings Case presentation, paragraph 2
2. Historical and current information from this episode of care organized as a timeline Case presentation, paragraph 2

**8a** Diagnostic testing (such as PE, laboratory testing, imaging, surveys). Case presentation, paragraph 2

**8b** Diagnostic challenges (such as access to testing, financial, or cultural) Case presentation, paragraph 2

**8c** Diagnosis (including other diagnoses considered) Case presentation, paragraph 2

**8d** Prognosis (such as staging in oncology) where applicable N/A

**9a** Types of therapeutic intervention (such as pharmacologic, surgical, preventive, self-care) Case presentation, paragraph 2

**9b** Administration of therapeutic intervention (such as dosage, strength, duration) Case presentation, paragraph 2

**9c** Changes in therapeutic intervention (with rationale) N/A

**10a** Clinician and patient-assessed outcomes (if available) Case presentation, paragraph 2

**10b** Important follow-up diagnostic and other test results Case presentation, paragraph 2

**10c** Intervention adherence and tolerability (How was this assessed?) Case presentation, paragraph 2

**10d** Adverse and unanticipated events Case presentation, paragraph 2

**Discussion 11a** A scientific discussion of the strengths AND limitations associated with this case report Discussion and conclusion, paragraph 1-4

**11b** Discussion of the relevant medical literature **with references** Discussion and conclusion, paragraph 5,6

**11c** The scientific rationale for any conclusions (including assessment of possible causes) Discussion and conclusion, paragraph 7

**11d** The primary “take-away” lessons of this case report (without references) in a one paragraph conclusion Discussion and conclusion, paragraph 8

**Patient Perspective 12** The patient should share their perspective in one to two paragraphs on the treatment(s) they received Case presentation, paragraph 2

**Informed Consent 13** Did the patient give informed consent? Please provide if requested . . . . . . . . . . . . . . . . . . . . . . . . . . . . . . . . . . . . . . **Yes ㇾ No**
